# Positron Emission Tomography in the Neuroimaging of Autism Spectrum Disorder: A Review

**DOI:** 10.3389/fnins.2022.806876

**Published:** 2022-04-13

**Authors:** Zhiqiang Tan, Huiyi Wei, Xiubao Song, Wangxiang Mai, Jiajian Yan, Weijian Ye, Xueying Ling, Lu Hou, Shaojuan Zhang, Sen Yan, Hao Xu, Lu Wang

**Affiliations:** ^1^Center of Cyclotron and PET Radiopharmaceuticals, Department of Nuclear Medicine and PET/CT-MRI Center, The First Affiliated Hospital of Jinan University, Guangzhou, China; ^2^Department of Rehabilitation Medicine, The First Affiliated Hospital of Jinan University, Jinan University, Guangzhou, China; ^3^Guangdong-Hongkong-Macau Institute of CNS Regeneration, Ministry of Education CNS Regeneration Collaborative Joint Laboratory, Jinan University, Guangzhou, China

**Keywords:** autism spectrum disorder, ASD, neuroimaging, positron emission tomography, PET, radioligands

## Abstract

Autism spectrum disorder (ASD) is a basket term for neurodevelopmental disorders characterized by marked impairments in social interactions, repetitive and stereotypical behaviors, and restricted interests and activities. Subtypes include (A) disorders with known genetic abnormalities including fragile X syndrome, Rett syndrome, and tuberous sclerosis and (B) idiopathic ASD, conditions with unknown etiologies. Positron emission tomography (PET) is a molecular imaging technology that can be utilized *in vivo* for dynamic and quantitative research, and is a valuable tool for exploring pathophysiological mechanisms, evaluating therapeutic efficacy, and accelerating drug development in ASD. Recently, several imaging studies on ASD have been published and physiological changes during ASD progression was disclosed by PET. This paper reviews the specific radioligands for PET imaging of critical biomarkers in ASD, and summarizes and discusses the similar and different discoveries in outcomes of previous studies. It is of great importance to identify general physiological changes in cerebral glucose metabolism, cerebral blood flow perfusion, abnormalities in neurotransmitter systems, and inflammation in the central nervous system in ASD, which may provide excellent points for further ASD research.

## Autism Spectrum Disorder

Autism spectrum disorder (ASD) is a pervasive developmental disorder characterized by persistently impaired social interactions and interpersonal communication, limited, repetitive, and stereotyped behaviors, and a significantly limited range of interests and activities. In 2013, the fifth edition of the Diagnostic and Statistical Manual of Mental Disorders (DSM-5) revolutionized the diagnostic criteria for ASD (American Psychiatric Association, 2013). In this manual, four categories previously defined as pervasive developmental disorders were identified: autistic disorder, Asperger syndrome (AS), pervasive developmental disorder not otherwise specified (PDD-NOS), and childhood disintegrative syndrome (also known as Heller Syndrome). These disorders are now collectively referred to as ASD. The diagnostic criteria were reduced from three items in DSM-IV to two in DSM-5: social dysfunction, repetitive and stereotypical behavior. Both verbal and non-verbal communication disorders are incorporated into social disorders. These shifts in diagnostic criteria have changed the diagnosis and treatment of different subtypes of pervasive developmental disorders, and affected the consistency and comparability of previous imaging studies. To avoid concept confusion, each cited document will be described with “Autism/AS/PDD/ASD” according to its diagnostic criteria in addition to ASD collectively.

The etiology of idiopathic ASD remains unclear and available evidence suggests the ASD phenotype is driven by complex interactions between genetic and environmental factors that affect brain maturation, synaptic function, and cortical networks. Environmental influences in early childhood development may play a key role in the development of ASD and obstetric complications may predispose vulnerable offspring to ASD (Brasic and Holland, 2006, 2007). At the same time, children who experience obstetric complications are also more likely to exhibit genetic causation of the disease (Katsanis, 2016). The latest large gene study has identified 102 risk genes associated with ASD in humans (Satterstrom et al., 2020). Most risk genes are expressed early in brain development and play a role in the regulation of gene expression or neuronal communication, thus having neurodevelopmental and neurophysiological affects. In cerebral cortex cells, risk genes are highly expressed in the spectrum of excitatory and inhibitory neurons, which is consistent with excitation/inhibition imbalances in multiple ASD-associated pathways.

At present, a diagnosis of ASD is mainly based on the medical history of patients and their families and clinical evaluation of patients. The two medical history data collection tools considered the gold standard for clinical diagnosis of ASD are: Autism Diagnostic Interview Revised (ADI-R), which is a structured interview for the family members of the patient (Le Couteur et al., 2003), and The Autism Diagnostic Observation Schedule (ADOS), which is a play-based interview with children and their families or with high-functioning children, adolescents, or adults (Lord et al., 2012). Clinical evaluation of patients with ASD includes physical and neurological examinations (Brasić et al., 2004). In addition, neuroimaging is also an effective evaluation tool for the diagnosis and treatment of ASD. Although there are currently no specific biomarkers available to diagnose ASD subtypes (i.e., without any known genetic or environmental causes), neuroimaging techniques have great potential for explaining the complexity of symptoms and signs observed in multiple ASD subtypes (Walton et al., 2013). In past decades, neuroimaging techniques have been used to explore the pathophysiologic mechanisms of ASD-related abnormalities in brain glucose metabolism, blood perfusion, and neurotransmitter systems. Neuroimaging techniques will play an increasingly important role in early diagnosis, treatment, and efficacy evaluation of ASD.

## Neuroimaging

Neuroimaging technology is used to visualize the structural and functional changes of the central nervous system. Structural imaging, including X-rays, computed tomography (CT) and magnetic resonance imaging (MRI), enables high-resolution visualization of anatomical structures. Functional imaging, including nuclear neuroimaging techniques such as positron emission tomography (PET) and single photon emission computed tomography (SPECT), as well as functional magnetic resonance (fMRI) techniques, help visualize and quantify functional and molecular-level changes in the nervous system (Wong et al., 2007). Non-invasive neuroimaging techniques provide dynamic and quantitative technical support for *in vivo* neuropsychiatric disorder studies, and are important means of studying structural, functional, and neurochemical molecule differences in the brains of ASD patients. The imaging techniques widely used in ASD research are MRI, SPECT, and PET.

### Magnetic Resonance Imaging

The combination of structural and functional MRI has significant advantages in the central nervous system. The main applied technologies in ASD research include magnetic resonance spectroscopy (MRS), blood oxygen-level dependent (BOLD) and arterial spin labeling (ASL). MRS can determine the molecular composition and spatial configuration of tissues by detecting the chemical shift of metabolites in magnetic resonance imaging. It can be applied to extract qualitative and quantitative information on various metabolites in the brain to characterize pathophysiological changes associated with brain diseases. The chemical shifts differ for different metabolites, which is a reflection of the pathological and physiological changes in different types of energy metabolism, including *n*-acetylaspartic acid (NAA), creatine (Cr) and creatine phosphate (Cr/PCr), adenosine diphosphate and triphosphate (ATP/ADP), choline (Cho), inositol (ml), glutamino-glutamate complex (Glx), and lactic acid (Wagner et al., 1985; Barker, 2001; Stanley, 2002; Page et al., 2006). BOLD and ASL can non-invasively detect changes in cerebral hemodynamics and brain function. It can display the activation state of cerebral cortex functional areas under task stimulation, which is of great value in locating the activated cortex and evaluating the brain function status, drug efficacy and prognosis of patients (Mash et al., 2019). fMRI has been widely used in animal experiments (Durieux et al., 2015; Janik et al., 2016; Mudd et al., 2017) and clinical trials (Tebartz van Elst et al., 2014; Goji et al., 2017) for ASD research.

### Single Photon Emission Computed Tomography

Single photon emission computed tomography (SPECT) are realized visualization by detecting the metabolism and distribution of single-photon radionuclide-labeled functional probes in the body, thus reflecting specific physiological changes. Probes with longer half-lives (*T*_1/2_) are commonly used clinically, such as ^123^I (*T*_1/2_ ∼13 h) and ^99m^Tc (*T*_1/2_ ∼24 h). Due to the high sensitivity of SPECT functional imaging and high resolution of CT structural imaging, SPECT/CT is an effective tool for *in vivo* disease diagnosis and new drug research (Van Audenhaege et al., 2015), and plays an important role in the neuroimaging of ASD (Makkonen et al., 2008; Bjørklund et al., 2018). Since SPECT and SPECT/CT are often available in nuclear medical departments of general hospitals, SPECT and SPECT/CT can typically be performed in community hospitals without cyclotrons.

### Positron Emission Tomography

Positron emission tomography (PET) detects processes associated with the metabolism and distribution of positron emitting radionuclide-labeled probes *in vivo*. Commonly used radionuclides include ^11^C (*T*_1/2_ ∼20.4 min), ^18^F (*T*_1/2_ ∼109.8 min), ^13^N (*T*_1/2_ ∼10.0 min) and ^15^O (*T*_1/2_ ∼2.0 min). It should be noted that radionuclides with short half-lives, such as ^11^C (*T*_1/2_ ∼20.4 min) and ^15^O (*T*_1/2_ ∼2.0 min), can only be conducted at tertiary medical centers with onsite cyclotrons. Simultaneously with metabolic distribution of the probe in the body, nuclear decay occurs and positrons emits. The positrons collide with surrounding electrons, and produce two gamma photons (511 keV each) in 180° reverse motion, which is recorded by multiple pairs of detectors located in opposite positions. Computer software then deal with the detected gamma photon signals to create three-dimensional images (Volkow et al., 1988). Compared to MRI and SPECT, PET is significantly more advantageous for the absolutely quantitative determination of parameters (such as glucose metabolic rate, protein synthesis rate, gene expression, enzyme activity and receptor density) related to specific biochemical pathways due to its advantages in sensitivity, spatial resolution and time efficiency.

Positron emission tomography imaging technology based on radioactive probe has unique technical advantages in tracer and quantitative detection of the distribution and change of various metabolites, receptors, enzymes, transporters, and study of drug absorption, excretion and other metabolic laws. Given the advantage of microdose, PET tracers can be used to conduct preclinical studies and early clinical trials (i.e., Phase 0 or early Phase I) to obtain target occupancy, pharmacokinetic and pharmacodynamic information, and preliminarily understand various physiological performance of drugs *in vivo*, providing a favorable basis for later clinical trials (McCluskey et al., 2020). PET probe is not only a diagnostic clinical drug, but also can be used as an evaluation tool for clinical translational research of other drugs. Over the course of the development of PET technology, more than 1,000 PET probes have been produced with many more being continuously and rapidly developed for disease research. There are a number of types of probes, including those used for imaging of metabolic substrates or analogs, such as glucose, amino acids, and fatty acids (Shiue and Welch, 2004), receptors using protein-binding probes, such as receptors and transporters (Smith et al., 2003; Gjedde et al., 2005), genes, immunological features, apoptosis, angiogenesis, hypoxia, and signal transduction (MacLaren et al., 2000; Jain and Batra, 2003). PET probes that have good brain penetration, high binding affinity and specificity, and a good metabolic stability and distribution volume are effective tools for neuroimaging. The main research targets of ASD and their corresponding PET probes are shown in Table 1.

**TABLE 1:** Summary of ASD research targets and their corresponding PET probes.

Target	PET probe	Chemical formula	Clinical application
Glucose	[^18^F]FDG	C_6_H_11_^18^FO_5_	Y
CBF	[^15^O]H2O	H_2_^15^O	Y
	[^15^O]CO2	CO^15^O	Y
	[^11^C]butanol	C_2_^11^CH_8_O	Y
**5-HT**			
Precursor	[^11^C]AMT	C_11_^11^CH_14_N_2_O_2_	Y
5-HTT	[^11^C](+)McN5652	C_18_^11^CH_21_NS	Y
	[^11^C]DASB	C_15_^11^CH_17_N_3_S	Y
	[^11^C]MADAM	C_15_^11^CH_20_N_2_S	Y
	[^18^F]FMeNER-d2	C_18_H_18_D_2_^18^FNO_3_	Y
	[^11^C]ADAM	C_14_^11^CH_17_IN_2_S	N
	[^11^C]DAPA	C_14_^11^CH_17_BrN_2_S	N
	[^11^C]AFM	C_15_^11^CH_19_FN_2_S	N
5-HT_2A_R	[^18^F]setoperone	C_21_H_24_^18^FN_3_O_2_S	Y
	[^11^C]MDL100907	C_22_H_18_^18^FNO_3_	Y
	[^18^F]altanserin	C_22_H_22_^18^FN_3_O_2_S	N
5-HT_1A_R	[^18^F]MPPF	C_25_H_27_^18^FN_4_O_2_	Y
	[^18^F]F13714	C_21_H_25_ClF^18^FN_4_O	N
**OXT**			
OXTR	[^11^C]PF-3274167	C_18_^11^CH_19_ClFN_5_O_3_	N
	[^11^C]EMPA	C_22_^11^CH_26_N_4_O_4_S	N
**DA**			
Precursor	[^18^F]FDOPA	C_9_H_10_^18^FNO_4_	Y
DAT	[^11^C]WIN35,428	C_15_^11^CH_20_FNO^2^	Y
	[^11^C]methylphenidate [^11^C]cocaine	C_14_H_19_NO_2_ C_17_H_21_NO_4_	N N
	[^18^F]FE-PE2I	C_20_H_25_^18^FINO_2_	N
D_2_R	[^11^C]NMS	C_23_^11^CH_28_FN_3_O_2_	Y
	[^11^C]raclopride	C_14_^11^CH_20_Cl_2_N_2_O_3_	Y
D_2_R,D_3_R	[^18^F]fallypride	C_20_H_29_^18^FN_2_O_3_	N
	[^11^C]-(+)-PHNO	C_14_^11^CH_21_NO_2_	N
D_1_R	[^11^C]NNC112	C_18_^11^CH_18_ClNO_2_	N
	[^11^C]SCH23390	C_16_^11^CH_18_ClNO	Y
**GABA**			
GABA_B_R	[^18^F]1b	C_16_H_16_Cl^18^FN_2_O_3_	N
GABA_A_R	[^18^F]FMZ	C_15_H_14_^18^FN_3_O_3_	Y
	[^11^C]Ro15-4513	C_14_^11^CH_14_N_6_O_3_	Y
**AChE**			
Receptor	[^18^F]FA	C_9_H_11_^18^FN_2_O	N
Precursor	[^11^C]MP4A	C_7_^11^CH_15_NO_2_	Y
Leucine	[^11^C]leucine	C_5_^11^CH_13_NO_2_	Y
**Glutamate**			
mGLuR5	[^18^F]FPEB	C_14_H_7_^18^FN_2_	Y
	[^11^C]ABP-688	C_14_^11^CH_16_N_2_O	N
mGLuR1	[^11^C]ITMM	C_18_^11^CH_18_N_5_O_2_S	N
mGLuR7	[^11^C]MMPIP	C_18_^11^CH_15_N_3_O_3_	N
TSPO	[^11^C]PK11195	C_20_^11^CH_21_ClN_2_O	Y
	[^11^C]DPA713	C_20_^11^CH_28_N_4_O_2_	N
	[^18^F]FEPPA	C_22_H_21_^18^FN_2_O_3_	N
	[^11^C]PBR28	C_20_^11^CH_20_N_2_O_3_	Y
	[^11^C]ER176	C_19_^11^CH_20_ClN_3_O	N
	[^18^F]GE180	C_20_H_27_^18^FN_2_O_2_	N
	[^18^F]FEPPA	C_22_H_21_^18^FN_2_O_3_	N
P2X7R	[^11^C]A-740003	C_25_^11^CH_30_N_6_O_3_	N
	[^11^C]JNJ-717	C_18_^11^CH_17_Cl_2_N_5_O_2_	N
	[^18^F]JNJ64413739	C_18_H_14_F_3_^18^FN_6_O	N
	[^11^C]SMW139	C_18_^11^CH_21_ClF_3_NO_2_	N
MAO-B	[^11^C]SL25.1188	C_15_^11^CH_17_F_3_N_2_O_5_	N
COX-1	[^11^C]PS13	C_17_^11^CH_16_F_3_N_3_O_3_	N
COX-2	[^11^C]MC1	C_16_^11^CH_17_N_3_O_3_S_2_	N
CSF1R	[^11^C]CPPC	C_21_^11^CH_27_N_5_O_2_	N
**AVP**			
V1aR	[^18^F]SRX246 [^11^C]SRX246 [^11^C](1S,5R)-1 [^11^C]PF-184563	C_42_H_48_^18^FN_5_O_5_ C_42_^11^CH_51_N_5_O_5_ C_25_^11^CH_30_N_2_O_2_ C_20_^11^CH_23_ClN_6_	N N N N

*Application, whether applied in ASD research; CBF, cerebral blood flow; 5-HT, 5-hydroxytryptamine/serotonin; 5-HTT, 5-hydroxytryptamine transporter/serotonin transporter; 5-HT_2A_R, serotonin 2A receptor; 5-HT_1A_R, serotonin 1A receptor; OXT, oxytocin; OXTR, oxytocin receptor; DA, dopamine; DAT, dopamine transporter; D_1_R, dopamine D1 receptor; D_2_R, dopamine D_2_ receptor; D_3_R, dopamine D_3_ receptor; GABA, γ-aminobutyric acid; GABA_A_R, γ-aminobutyric acid type A receptor; GABA_B_R, γ-aminobutyric acid type B receptor; AChE, acetylcholinesterase; mGLuR1, metabotropic glutamate receptor 1; mGLuR5, metabotropic glutamate receptor 5; mGLuR7, metabotropic glutamate receptor 7; TSPO, 18 kDa translocator protein; P2X7R, purinergic P2X7 receptor; MAO-B, monoamine oxidase B; COX-1, cyclooxygenase 1; COX-2, cyclooxygenase 2; CSF1R, colony stimulating factor 1 receptor; AVP, arginine vasopressin; V1Ar, vasopressin 1a receptor; Y, yes; N, no.*

## Positron Emission Tomography Molecular Imaging in Autism Spectrum Disorder

The development of PET has provided non-invasive and dynamic technical support for the study of ASD *in vivo* (Syed and Brasic, 2019). Although the etiology and pathology of ASD are complex and unclear, PET has been used by researchers over past decades to explore the pathophysiological changes in ASD from various angles, resulting in a large amount of evidence being obtained and new research directions opening up (Schifter et al., 1994; Rumsey and Ernst, 2000; Chugani, 2012; Zürcher et al., 2015; Hwang et al., 2017; Wolff et al., 2018; Girault and Piven, 2020; Meyer et al., 2020; Kowalewska et al., 2021).

The neuroimaging studies on ASD have been comprehensively summarized in several literatures. Due to the difference of time scale and research angle, the contents and focus in each review are different. Early published literature, such as the comprehensive systematic review by Zürcher et al. (2015), provided us with valuable references, but now a large number of new research results need to be supplemented. Some of the reviews published in recent years have focused on summarizing the state of ASD imaging research in general. For example, McPartland et al. (2021) recently published a review about “Looking Back at the Next 40 Years of ASD Neuroscience Research,” which discussed the most comprehensive analysis of the five most common neuroscience modalities employed in ASD research. Kowalewska et al. (2021) only stated consistent results from ASD studies by screening journal with high impact factors. Others, while covering several neuroimaging techniques, tend to focus on the application results of a specific neuroimaging technique in detail, such as Li et al. (2021) discuss more about MRI content.

As the foundation work of ASD PET probe development, and the need to constantly update and summarize the existing research results. The authors conducted a PubMed, EMBASE, Scopus and PsychINFO search of ASD PET literature published before 2022.01.01 using various combinations of “ASD,” “autism spectrum disorder” and “PET.” In this paper, we discuss cerebral glucose metabolism, cerebral blood perfusion, neurotransmitter system (precursors, transporters and receptors) and some new research targets, such as neuroinflammation related targets and arginine vasopressin receptor targets. By summarizing the clear pathophysiological mechanism of ASD and stating the unclear research conclusions, the application status and prospect of PET technology in ASD research were discussed. It provides a more comprehensive and detailed literature review for researchers in the field of ASD PET probe development, which is helpful for readers to grasp the *status quo* of ASD PET research more intuitively and conveniently, and hopefully provides some enlightenment for readers to explore new research directions.

### Cerebral Glucose Metabolism

Glucose is the main energy source of brain cells and thus the state of glucose metabolism partly reflects changes in brain function. As a glucose analog, [^18^F]FDG can be taken up by brain cells that utilize high levels of glucose and undergo specific intracellular phosphorylation. Prior to radioactive decay, [^18^F]FDG is unable to complete intracellular glycolysis due to the loss of oxygen at site 2 and, therefore, imaging using [^18^F]FDG is a good reflection of glucose uptake and phosphorylation distribution in brain cells (Fowler and Ido, 2002). [^18^F]FDG is very valuable in ASD PET research due to its excellent imaging characteristics, including a long half-life, short scanning time, mature preparation process, and relatively simple scanning process (Zürcher et al., 2015).

The subject of brain glucose metabolism changes is among the most widely studied in ASD PET. The majority of experimental studies focus on differences in brain glucose metabolism between ASD patients and normal subjects. However, due to the differences in experimental subjects and design, there remains no consensus on changes of FDG metabolism in individuals with ASD. As early as 1985, Rumsey et al. (1985) conducted a brain [^18^F]FDG PET study on 10 adult males with autism and 15 typical developing controls, and found the cerebral glucose metabolism of the patients with autism was more diffuse. However, most [^18^F]FDG PET studies since then have found brain glucose metabolism in ASD is typically reduced in local brain tissue compared to typical developing controls; however, this is not true for specific brain regions (Buchsbaum et al., 1992; Chugani et al., 1996; Haznedar et al., 1997, 2000, 2006; Deriaz et al., 2012; Dilber et al., 2013; Manglunia and Puranik, 2016). In these cases, Chugani et al. (2007) conducted a [^18^F]FDG PET study on four autistic children with wine spotting, and decreased glucose metabolism in the bilateral medial temporal regions, anterior cingulate gyrus, frontal cortex, right temporal cortex, and cerebellum was observed. The frontal temporal lobe glucose metabolism asymmetry was opposite that of the typical child with autism. In a few studies, patients with ASD displayed both decreased and increased glucose metabolism in different brain regions (Siegel et al., 1992; Asano et al., 2001; Hazlett et al., 2004; Anil Kumar et al., 2017; Mitelman et al., 2018; Kadwa et al., 2019). In addition, earlier studies also reported no significant difference in cerebral glucose metabolism between the autism and control subjects (De Volder et al., 1987; Herold et al., 1988; Heh et al., 1989). As summarized in Table 2, there is no consensus on decreases or increases in glucose metabolism in specific brain regions in patients with ASD, however, the most common discoveries in brain glucose metabolism patterns in patients with ASD were decreases in glucose metabolism in the temporal lobe and abnormal metabolism in areas highly connected to the temporal lobe, such as the frontal lobe, parietal lobe, and adjacent limbic cortex. This is consistent with the anatomical connectivity between the supratemporal and associative cortexes (Seltzer and Pandya, 1978; Pandya and Yeterian, 1985; Sitoh and Tien, 1997) and is supported by findings on the brain functional network for glucose metabolism (Lee et al., 2011a,b, 2012).

**TABLE 2:** FDG PET studies in ASD.

Citation	Target	Tracer	Subject (*n*) Sex (M/F)	Age (year): Mean ± SD, range	WAIS scores IQ: mean ± SD, range	Diagnosis	Conclusions
Rumsey et al., 1985	Glucose	FDG	ASD: *n* = 10(M) CON: *n* = 15(M)	ASD: 26 ± 6, 18–36 CON: 28 ± 5, 20–37	VIQ: 91 ± 23, 48–117 PIQ: 96 ± 23, 55–126	Autism [DSM-III]	Hypermetabolism in whole brain of ASD.
De Volder et al., 1987	Glucose	FDG	ASD: *n* = 11(M)/7(F) CON *n* = 15/3/3(–)	ASD: 10 ± 4, 2–18 CON: 22/7–15/9–12	–	Autism [DSM-III]	No differences between groups.
Herold et al., 1988	Glucose	FDG	ASD: *n* = 6(M) CON: *n* = 6(M)/2(F)	ASD: 22.2, 21–25 CON: 34, 22–53	FSIQ: <45–92 VIQ: <45–91 PIQ: <45–99	Autism [DSM-III-R, ICD-10]	No differences between groups.
Horwitz et al., 1988	Glucose	FDG	ASD: *n* = 14(M) CON: *n* = 14(M)	ASD: 27 ± 7, 18–39 CON: 27 ± 7	VIQ: 93 ± 21, 48–117 PIQ: 99 ± 21, 55–126	Autism [DSM-III]	Fewer positive correlation between rGMRglu in parietal lobe and whole brain of ASD.
Heh et al., 1989	Glucose	FDG	ASD: *n* = 5(M)/2(F) CON: *n* = 7(M)/1(F)	ASD: 23 ± 6, 19–36 CON: 24 ± 5, 20–35	VIQ: 91 ± 25, 72–131 PIQ: 94 ± 19, 75–132	Autism [ICDS, DSM-III]	No differences in cerebellum between groups.
Buchsbaum et al., 1992	Glucose	FDG	ASD: *n* = 5(M)/2(F) CON: *n* = 13(M)	ASD: 25 ± 3, 19–38 CON: 24 ± 6	VIQ: 930 ± 16, 78–122; PIQ: 90 ± 13, 75–112	Autism [DSM-III, ICDS, K-SADS, DICA]	Hypometabolism in right posterior thalamus and right putamen of ASD.
Siegel et al., 1992	Glucose	FDG	ASD: *n* = 12(M)/4(F) CON: *n* = 19(M)/7(F)	ASD: 23 ± 6, 17–38 CON: 27 ± 7	IQ: 90 ± 17, 74–135	Autism [ICDS]	Hypometabolism in left posterior putamen and hypermetabolism in right posterior calcarine cortex of ASD.
Schifter et al., 1994	Glucose	FDG	ASD: *n* = 9(M)/4(F)	ASD: 7 ± 2, 4–11	–	Autism [DSM-III-R]	Abnormal rGMRglu in 4/13 ASD patients.
Siegel et al., 1995	Glucose	FDG	ASD: *n* = 12(M)/3(F) CON: *n* = 13(M)/7(F)	ASD: 24 ± 7, 17–38 CON: 25 ± 6, 19–39	–	Autism [ICDS]	Negative correlation between rGMRglu in medial frontal cortex and attentional performance.
Chugani et al., 1996	Glucose	FDG	ASD: *n* = 10(M)/8(F) CON: *n* = 10(–)	ASD: 2 ± 1, 1–5	–	Autism [DSM-IV]	Children with infantile spasms and temporal hypometabolism more likely to be autistic.
Haznedar et al., 1997	Glucose	FDG	ASD: *n* = 5(M)/2(F) CON: *n* = 7(–)	ASD: 24 ± 11, 17–47 CON: 26 ± 9, 20–47	IQ: 60–125	Autism [ADI]	Hypometabolism in AC of ASD.
Haznedar et al., 2000	Glucose	FDG	ASD: *n* = 15(M)/2(F) CON: *n* = 15(M)/2(F)	ASD: 28 ± 11, 17–55 CON: 29 ± 9, 20–56	IQ: 55–125	Autism or AS [DSM-IV]	Hypometabolism in AC and posterior cingulate of ASD.
Asano et al., 2001	Glucose	FDG	ASD: *n* = 9(–) CON *n* = 9/8(–)	ASD: 4 ± 3 CON: 6 ± 2/6 ± 3	–	Autism [ADI-R, GARS, VABS]	Hypometabolism in lateral temporal gyri bilaterally and hypermetabolism in cerebellar nuclei bilaterally of ASD.
Buchsbaum et al., 2001	Glucose	FDG	ASD: *n* = 5(M)/1(F)	ASD: 31 ± 9	IQ: 95 ± 26, 53–119	Autism or AS [DSM-IV, ADI]	Hypermetabolism in AC, orbitofrontal areas and striatum of ASD after fluoxetine treatment.
Hazlett et al., 2004	Glucose	FDG	ASD: *n* = 15(M)/2(F) CON: *n* = 15(M)/2(F)	ASD: 28 ± 11, 17–55 CON: 29 ± 9, 20–56	IQ: 93 ± 26, 55–135	Autism or AS [DSM-IV]	Hypometabolism in medial/cingulate areas and hypermetabolism in occipital and parietal areas of ASD.
Haznedar et al., 2006	Glucose	FDG	ASD: *n* = 15(M)/2(F) CON: *n* = 15(M)/2(F)	ASD: 28 ± 11, 17–55 CON: 29 ± 9, 20–56	IQ: 97 ± 25, 55–125	Autism or AS [DSM-IV]	Hypometabolism bilaterally in ventral caudate, putamen and thalamus of ASD.
Chugani et al., 2007	Glucose	FDG	Port-wine stain: *n* = 2 (M)/2(F) ASD: *n* = 12(–)	Port-wine stain: 3–6 ASD: 3–8	–	Autism [ADI-R, AQ, VABS, GARS]	Port-wine stain + ASD: Hypometabolism in cerebellum, medial temporal, AC, lateral temporal and frontal cortices. ASD: Hypometabolism in lateral temporal cortex and hypermetabolism in AC and medial temporal cortex.
Lee et al., 2011a	Glucose	FDG	ASD: *n* = 26(–) ADHD: *n* = 24(–) CON: *n* = 11(–)	ASD: 6 ± 2 ADHD: 8 ± 2 CON: 10 ± 3	–	ASD [Korean ADI-R]	Segmented brain connectivity and temporal lobe asymmetry in ASD.
Lee et al., 2011b	Glucose	FDG	ASD: *n* = 26(–) CON: *n* = 11(–)	ASD: 6 ± 2 CON: 10 ± 3	–	ASD [Korean ADI-R]	Local overconnectivity and long-range underconnectivity in ASD.
Deriaz et al., 2012	Glucose	FDG	ASD: *n* = 1(M)	ASD: 20	–	PDD w/SIB [Clinical evaluation]	Hypometabolism in right temporo-parietal area and right left posterior fossa of ASD.
Lee et al., 2012	Glucose	FDG	ASD: *n* = 26(–) ADHD: *n* = 24(–) CON: *n* = 11(–)	ASD: 6 ± 2 ADHD: 8 ± 2 CON: 10 ± 3	–	ASD [Korean ADI-R, ADOS]	Atypical connectivity between left inferior prefrontal regions and other brain areas in ASD.
Dilber et al., 2013	Glucose	FDG	ASD: *n* = 9(M)/6(F) CON: *n* = 6(M)/3(F)	ASD: 9 ± 4, 3–16 CON: 7 ± 4, 3–14	–	Autism [DSM-IV, ABC, CARS]	Hypometabolism in temporal lobe (13/15), frontal lobe (9/15) and parietal lobe (7/15) of ASD.
Sharma et al., 2013	Glucose	FDG	ASD: *n* = 24(M)/8(F)	ASD: 11 ± 6, 3–33	–	Autism [DSM-IV-TR]	Abnormal rGMRglu in frontal, temporal, parietal, occipital lobes, cerebellum, cingulate, amygdala, hippocampus, and basal ganglia of ASD.
Manglunia and Puranik, 2016	Glucose	FDG	ASD: *n* = 1(–)	ASD: 6	–	Autism [DSM-IV, ABC, CARS]	Hypometabolism in temporal lobes of ASD.
Park et al., 2017	Glucose	FDG	ASD: *n* = 1(M)	ASD: 14	IQ: 60 (8 years old)	ASD [GCI, ABC, CY-BOCS, K-ARS, SRS]	Hypometabolism in prefrontal and frontal cortex of ASD after NAc DBS.
Anil Kumar et al., 2017	Glucose	FDG	ASD: *n* = 9(M)/1(F) CON: *n* = 13(M)/2(F)	ASD: 8–19	IQ: 79 ± 10	Autism [DSM-IV]	Hypometabolism in cerebellum and temporal lobes and hypermetabolism in frontal and occipital lobes of ASD.
Mitelman et al., 2018	Glucose	FDG	ASD: *n* = 4(M)/21(F) schizophrenia: *n* = 9(M)/32(F) CON: *n* = 26(M)/25(F)	ASD: 32 ± 12 Schizophrenia: 40 ± 18 CON: 33 ± 13	VIQ: 110 ± 21 PIQ: 106 ± 20	Autism [DSM-IV, ADI-R]	Hypometabolism in parietal lobe, frontal premotor, eye-fields area, amygdala and hypermetabolism in posterior cingulate, occipital cortex, hippocampus, basal ganglia of ASD.
Żarnowska et al., 2018	Glucose	FDG	ASD: *n* = 1(M)	ASD: 6	IQ: 82	Autism [DSM-IV-TR]	Hypometabolism in whole brain of ASD after KD.
Kadwa et al., 2019	Glucose	FDG	ASD: *n* = 85(M)/15(F) CON: *n* = 100(–)	ASD: 3–12	–	Autism [DSM-V, CARS]	Hypometabolism in diffuse cerebral/temporal and parietal lobe and hypermetabolism in bilateral frontal lobe of ASD.

*AC, anterior cingulate; ADI, Autism Diagnostic Interview; ADI-R, ADI-revised; ADOS, Autism Diagnostic Observation Schedule; AS, Asperger syndrome; ASD, autism spectrum disorder; AQ, Autism Spectrum Quotient; CARS, Childhood Autism Rating Scale; DICA, Diagnostic Interview for Children and Adolescents; DSM-III, Diagnostic and Statistics Manual of Mental Disorder Third Edition; DSM-III-R, DSM Third Edition Revised; DSM-IV, DSM Fourth Edition; DSM-IV-TR, DSM Fourth Edition Text Revised; FSIQ, Full Scale Intelligence Quotient; GARS, Gilliam Autism Rating Scale; ICDS, Interview for Childhood Disorders and Schizophrenia; ICD-10, International Classification of Diseases Tenth Revision; IQ, Intelligence Quotient; K-SADS, Kiddie Schizophrenia and Affective Disorder Scales; PDD, Pervasive Developmental Disorder; PIQ, Performance Intelligence Quotient; rGMRglu, relative glucose metabolic rate; VABS, Vineland Adaptive Behavior Scale; VIQ, Verbal Intelligence Quotient; GCI, Clinical Global Impairment; CY-BOCS, Children’s Yale-Brown OC Scale; K-ARS, Korean ADHD Rating Scale; SRS Social Responsiveness Scale.*

[^18^F]FDG PET is not only used in the study of glucose metabolism changes in specific brain regions in patients with ASD, but also in evaluation by neuroimaging before and after ASD treatment. An [^18^F]FDG PET study conducted on five adult patients with autism and one with AS before and after fluoxetine treatment found the glucose metabolic rate in the right frontal lobe, especially in the anterior cingulate gyrus and orbitofrontal cortex, was significantly higher after treatment than before. In addition, patients who presented with higher glucose metabolic rates in the medial frontal lobe and anterior cingulate before treatment were more likely to respond well to fluoxetine, suggesting responses to fluoxetine can be predicted based on baseline cingulate metabolism (Buchsbaum et al., 2001). However, cerebral glucose metabolism after ASD treatment does not always show an increase in glucose metabolism. A [^18^F]FDG PET study in a 14-year-old boy with ASD assessing before and after bilateral deep brain stimulation (DBS) in the nucleus accumbens (NAc) found the patient’s clinical symptoms improved significantly after 2 years of treatment with NAc DBS. Brain [^18^F]FDG PET results showed glucose metabolism in the prefrontal, frontal, and occipital cortexes was significantly lower than before treatment (Park et al., 2017). Another [^18^F]FDG PET study assessing before and after treatment with a ketogenic diet (KD), which is a mature therapy for refractory epilepsy, in a 6-year-old child with ASD revealed bilateral local reductions in glucose metabolism in the proximal meso-temporal lobe, basal ganglia region, and cerebellum in patients with ASD. After 12 months on a KD, the patient’s glucose metabolism had decreased significantly and diffusely across the entire cerebral cortex compared to before KD (Żarnowska et al., 2018). The underlying reason for this result is the conversion of the main energy source of the brain cells from glucose to ketone bodies (Frye and Rossignol, 2014; Courchesne-Loyer et al., 2017). These findings suggest ASD therapy can improve clinical symptoms and alter the state of cerebral glucose metabolism in patients with ASD. The use of [^18^F]FDG PET to assess glucose metabolism levels before and after ASD treatment can not only help in effectively screening treatment subjects before clinical treatment, but also provide support for the evaluation of efficacy after clinical treatment. In addition, the effects of ASD drugs acting on 5-hydroxytryptamine, dopamine, gamma-aminobutyric acid, and other neurotransmitter systems with influence on brain glucose metabolism should be further explored.

### Cerebral Blood Flow

The [^15^O]CO_2_ steady-state inhalation technique was used in the early stages of PET imaging to study cerebral perfusion, but its reliability is insufficient. Imaging with probes that have a small relative molecular weight and are uncharged and fat-soluble (mainly [^15^O]H_2_O) are now more preferable. These probes can enter brain cells through the blood–brain barrier, where the amount of probe entering brain cells is positively correlated with regional cerebral blood flow (rCBF). Because rCBF generally directly correlates with metabolism associated with local brain function, cerebral perfusion imaging can also reflect the state of local brain function to a certain extent. To measure rCBF, it is necessary to record the dynamic intracranial distribution of the probe after the probe enters the human body. Meanwhile, blood samples of the subjects are continuously collected to evaluate carotid artery input function. With the help of image processing, radiation concentration-time curves are obtained to calculate the rCBF value of the corresponding brain region (Joseph-Mathurin et al., 2018).

In early experiments, cerebral perfusion imaging was performed on six patients with autism and eight normal controls after nasal inhalation of [^15^O]CO_2_ and no difference in rCBF was found (Herold et al., 1988). These negative results may be due to the influence of [^15^O]CO_2_ absorption on cerebral perfusion imaging quality or limitations of early low-resolution PET cameras. High-resolution PET combined with statistical parameter mapping was able to find some local abnormalities which the low-resolution PET failed to detect (Boddaert et al., 2002). In subsequent experiments, intravenous injection of [^15^O]H_2_O was used for cerebral perfusion imaging on ASD patients. These researches conducted cerebral perfusion imaging while patients carried out various social functions with related tasks (such as emotional processing, theory of mind, and listening and speaking skills), and then image data analysis based on the statistical parameters of voxel was used to compare perfusion in distinct brain regions in stimulated patients with ASD and normal controls (Happé et al., 1996; Müller et al., 1998; Hall et al., 2003). According to different activation tasks, differences in the activation of regions based on the cerebral perfusion imaging results were usually related to behavioral functional control regions of the human brain. As the ability of patients with ASD to perform tasks varies greatly due to individual factors, such as age and IQ, the results of different experiments were not comparable (Table 3). However, some continuous studies have revealed rules for changes in brain function and rCBF in ASD. When [^15^O]H_2_O PET was used to study differences in rCBF in four adult patients with autism and five typical developing controls during a verbal task (Müller et al., 1998), the right dentate nucleus and left frontal region 46 were activated less during speech, hearing, and expressive language, and more during motor speech function in those with autism. The intergroup differences in the thalamus were consistent with those in region 46 during speech expression. In 1999, [^15^O]H_2_O PET was used to explore differences in rCBF between five highly functioning adult patients with autism and five typical developing controls during a verbal task (Müller et al., 1999). The results partially support atypical (diminished or reversed) hemispheric dominance of receptive language. In the patient group, hearing was associated with a significant reversal of normal left hemisphere dominance, while decreases in the bilateral superior temporal gyrus and cerebellar rCBF in those with autism suggests a decrease in cerebellar involvement in non-verbal auditory perception and possible expression of language. These results may indicate atypical functional specialization of the dentate-thalamo-cortical pathway and are consistent with a regional-specific biochemical disorder model of the brain in development of autism. This is in line with previous findings that dys-serotonin synthesis affects the dentate-thalamo-cortical pathway in boys with autism (Chugani et al., 1997). Three experiments using [^15^O]H_2_O to evaluate differences in rCBF between patients with autism and normal controls during a language task confirmed abnormal functioning of the temporal lobe region with decreased rCBF (mainly in the left temporal region) in those with autism, which is consistent with changes in the glucose metabolism pattern in brains with autism (Boddaert et al., 2002, 2003, 2004).

**TABLE 3:** CBF PET studies in ASD.

Citation	Target	Tracer	Subject (*n*) Sex(M/F)	Age (year): mean ± SD, range	WAIS scores IQ: mean ± SD, range	Diagnosis	Condition	Conclusions
Herold et al., 1988	CBF	[^15^O]CO_2_	ASD: *n* = 6(M) CON: *n* = 4(M)/2(F)	ASD: 22, 21–25 CON: 25, 22–29	–	Autism [DSM-III-R, ICD-10]	Rest	No differences between groups.
Happé et al., 1996	CBF	[^15^O]H_2_O	ASD: *n* = 5(M) CON: *n* = 6(–)	ASD: 24, 20–27 CON: –	FISQ: 100, 87–112 VIQ: 110, 93–125 PIQ: 92, 83–100	AS [Clinical evaluation]	Phys ToM US	Increased rCBF in more ventral mPFC and abnormal rCBF in left mPFC of ASD.
Fernell et al., 1997	CBF	[^15^O]H_2_O	ASD: *n* = 4(M)/2(F)	ASD: 3–5	DQ: 20–70	Autism [DSM-III-R and CARS]	Rest	No rCBF change after R-BH4 treatment.
Müller et al., 1998	CBF	[^15^O]H_2_O	ASD: *n* = 4(M) CON: *n* = 5(M)	ASD: 26, 18–31 CON: 26, 23–30	FSIQ: 77 ± 10, 71–92 VIQ: 82 ± 16, 71–105 PIQ: 75 ± 8, 67–84	Autism [DSM-IV, GARS]	(i) Rest (ii) Listening to sentences (iii) Repeating sentences (iv) Generating sentences	Decreased rCBF (auditory and language tasks) and increased rCBF (motor speech tasks) in right dentate nucleus and left frontal BA46.
Müller et al., 1999	CBF	[^15^O]H_2_O	ASD: *n* = 4(M)/1(F) CON: *n* = 5(–)	ASD: 27, 18–31 CON: 28, 23–34	FSIQ: 76 ± 9, 69–92 VIQ: 81 ± 14, 71–105 PIQ: 74 ± 7, 67–84	Autism [GARS]	(i) Rest (ii) Listening to sentences (ii) Listening to sentences (iv) Repeating sentences (v) Generating sentences	Language perception: Reversal of left hemispheric dominance. Non-verbal auditory perception: decreased rCBF in cerebellum.
Zilbovicius et al., 2000	CBF	[^15^O]H_2_O	ASD(i): *n* = 17(M)/4(F) ASD(ii): *n* = 11(M)/1(F) CON: *n* = 8(M)/2(F)	ASD(i):8 ± 3, 5–13 ASD(ii):7 ± 2, 5–13 CON: 8 ± 2, 5–13	–	Autism [DSM-IV]	Rest	Decreased rCBF in bilateral temporal lobes of ASD.
Boddaert et al., 2002	CBF	[^15^O]H_2_O	ASD: *n* = 17(M)/4(F) CON: *n* = 8(M)/2(F)	ASD: 8 ± 3, 5–13 CON: 8 ± 2, 5–13	–	Autism [DSM-IV]	Rest	Decreased rCBF in STS, STG and bilateral temporal lobe of ASD.
Castelli et al., 2002	CBF	[^15^O]H_2_O	ASD: *n* = 10(–) CON: *n* = 10(–)	ASD: 33 ± 8 CON: 25 ± 5	–	Autism or AS [DSM-IV]	ToM GD Rd animation	Mentalizing-triggering animations: decreased rCBF in mPFC, STS and temporal poles.
Boddaert et al., 2003	CBF	[^15^O]H_2_O	ASD: *n* = 4(M)/1(F) CON: *n* = 8(M)	ASD: 19 ± 5 CON: 22 ± 3	IQ: 64 ± 5	Autism [DSM-IV, ADI-R]	(i) Rest (ii) Listening to speech (iii) Listening to speech	Listening to speech: decreased rCBF in left temporal areas and increased rCBF in right middle frontal gyrus.
Hall et al., 2003	CBF	[^15^O]H_2_O	ASD: *n* = 8(M) CON: *n* = 8(M)	ASD: 20–33	NVIQ: 105 ± 18, 80–130	Autism or AS [DSM-IV]	(i) Emotion-recognition task (ii) Gender-recognition baseline task	Emotion-recognition task: decreased rCBF in inferior frontal, fusiform areas and increased rCBF in right anterior temporal pole, AC, thalamus.
Nieminen-von Wendt et al., 2003	CBF	[^15^O]H_2_O	ASD: *n* = 8(M) CON: *n* = 8(M)	ASD: 28 ± 6, 19–38 CON: 32 ± 5, 23–40	FIQ:109 ± 16, 88–140 VIQ:108 ± 15, 82–131 PIQ:111 ± 167, 95–144	AS [DSM-IV, ICD-10]	Phys ToM	Increased rCBF in cerebellum of ASD.
Boddaert et al., 2004	CBF	[^15^O]H_2_O	ASD: *n* = 10(M)/1(F) CON: *n* = 4(M)/2(F)	ASD: 7 ± 2, 4–10 CON: 7 ± 2, 3–9	IQ: 43 ± 21	Autism [DSM-IV, ADI-R]	(i) Rest (ii) Listening to speech (iii) Listening to speech	Decreased rCBF in left middle temporal gyrus and precentral frontal gyrus of ASD.
Boddaert et al., 2005	CBF	[^15^O]H_2_O	ASD: *n* = 1(M)	20	IQ: 66	Autism [DSM-IV, ADI-R]	(i) Calendar task (ii) Word repetition control task	Calendar task: increased rCBF in left hippocampus, left frontal cortex and left middle temporal lobe.
Gendry Meresse et al., 2005	CBF	[^15^O]H_2_O	ASD: *n* = 37(M)/8(F)	ASD: 8 ± 2, 5–11.9	IQ: 44 ± 22	Autism [DSM-IV, ADI-R]	Rest	Negative correlation between rCBF and ADI-R score in left STG.
Duchesnay et al., 2011	CBF	[^15^O]H_2_O	ASD: *n* = 37(M)/8(F) CON: *n* = 9(M)/4(F)	ASD: 8 ± 2, 5–12 CON: 8 ± 2, 5–12	IQ: 45 ± 22 DQ: 44 ± 23	ASD [DSM IV-R, ADI-R]	Rest	Decreased rCBF in right STS and increased rCBF in contralateral post central area of ASD.
Pagani et al., 2012	CBF	[^11^C]butanol	ASD: *n* = 6(M)/5(F) CON: *n* = 5(M)/5(F)	ASD: 32 ± 9, 20–48 CON: 29 ± 8, 20–42	FSIQ:104 ± 17, 87–135 VIQ:105 ± 16, 83–133 PIQ:102 ± 18, 82–134	Autism [DSM-IV, GAF, ADOS-G, RAADS-R, AQ]	Rest	Increased rCBF in para-hippocampal, posterior cingulate, primary visual and temporal cortex, putamen, caudate, substantia nigra and cerebellum.

*ADI, Autism Diagnostic Interview; ADI-R, ADI-revised; AS, Asperger syndrome; ASD, autism spectrum disorder; CBF, cerebral blood flow; AQ, Autism Spectrum Quotient; DQ, Developmental Quotient; DSM-III-R, Diagnostic and Statistics Manual of Mental Disorder Third Edition Revised; DSM-IV, DSM Fourth Edition; FISQ, Full Scale Intelligence Quotient; ICD-10, International Classification of Diseases Tenth Revision; IQ, Intelligence Quotient; NVIQ, Non-verbal Intelligence Quotient; PFC, prefrontal cortex; PIQ, Performance Intelligence Quotient; rCBF, regional cerebral blood flow; STG, superior temporal gyrus; STS, superior temporal sulcus; VIQ, Verbal Intelligence Quotient; Phys, physical stories; ToM, stories requiring mentalizing; US, unconnected sentences; GD, goal-direction action; Rd, random.*

For task-state cerebral perfusion imaging, functional magnetic resonance such as arterial spin labeling (ASL) sequence has unique advantages (Saitovitch et al., 2019). In the future, joint imaging or comparative studies with PET and fMRI will have more widespread application prospects.

### 5-Hydroxytryptamine System

5-Hydroxytryptamine (5-HT), also known as serotonin, is an inhibitory neurotransmitter that regulates neuronal migration and synaptogenesis during brain development (Yang et al., 2014). Abnormalities of the 5-HT system in autism were first reported in 1961 (Schain and Freedman, 1961). Whole blood tests in 23 children with autism and severe mental disability showed 5-HT was significantly increased, as well as slightly increased in seven children with severe mental disability without autism. Four non-autistic and non-mentally handicapped children and 12 children with borderline or mild mental retardation had normal whole blood 5-HT concentrations. Follow-up studies confirmed the elevated whole blood 5-HT levels in patients with autism, and found that immediate family members of the patient also had elevated whole blood 5-HT levels (Hwang et al., 2017). In addition, 5-HT antagonists have been shown to lead to some improvement in autism-related symptoms, although not the core symptoms associated with autism (McDougle et al., 1996; Croen et al., 2011; Hollander et al., 2012). Given the overwhelming evidence of the association between the 5-HT system and ASD, 5-HT has become the most common neurotransmitter system assessed in studies of ASD. To summarize the current ASD-related 5-HT PET imaging studies, abnormalities of the 5-HT system in patients with ASD mainly manifest as an increase in 5-HT concentration in peripheral blood, disorder of 5-HT synthesis in the brain, a decrease in 5-HT transporters in the brain, and abnormal functioning of 5-HT receptors in the brain.

#### 5-Hydroxytryptamine Precursor

Tryptophan hydroxylase is a precursor of 5-HT and α-methyl-L-tryptophan can specifically bind to tryptophan hydroxylase. Therefore, radio-labeled [^11^C]AMT has been widely used as a specific probe to measure 5-HT synthesis (Chakraborty et al., 1996; Chugani and Muzik, 2000). Unilateral changes in 5-HT synthesis in the dentate-thalamo-cortical pathway in boys with autism were analyzed with [^11^C]AMT PET (Chugani et al., 1997). Asymmetries in 5-HT synthesis were found in the frontal cortex, thalamus, and cerebellar dentate nucleus in all seven boys tested, but not in a girl with autism. Five of the boys with autism had reduced 5-HT synthesis in the left frontal cortex and thalamus, while the other two had reduced 5-HT synthesis in the right frontal cortex and thalamus. In all seven patients, elevated 5-HT synthesis was observed in the contralateral dentate nucleus. In addition, Chugani et al. (1999) also used [^11^C]AMT PET to measure 5-HT synthesis in 30 children of different ages with autism, 8 of their non-autistic siblings, and 16 non-autistic children with epilepsy and the mechanism underlying abnormal 5-HT synthesis in autism was further explored. The results showed that 5-HT synthesis in a non-autistic child before the age of 5 was more than twice that of an adult and then decreased to adult levels after the age of 5. However, whole-brain 5-HT synthesis decreased during childhood in children with autism, but increased gradually between the ages of 2 and 15 years, where it reached 1.5 times the normal adult value and showed no gender difference. There were significant age-dependent differences in 5-HT synthesis between the autism and epileptic groups, and the autism and sibling groups. Chandana et al. (2005) used [^11^C]AMT PET to explore the association between global and local abnormalities in serotonin synthesis and handedness and language function in children with autism. By measuring cerebral 5-HT synthesis in 117 children with autism, multiple abnormal cortical involvement patterns, including in the right cortex, left cortex, and non-lateralization, were observed. Children with autism who have reduced AMT binding in the left cortex showed greater severe language impairment, while there was a higher autism prevalence with reduced AMT binding in the right cortex among those that were left-handed and ambidextrous. Abnormal asymmetrical development of the serotonin system both globally and locally may result in faulty neural circuitry that specifies hemispheric specialization.

#### 5-Hydroxytryptamine Transporter

The mechanism by which the human body regulates the 5-HT concentrations outside of brain cells is mainly through 5-hydroxytryptamine transporter (5-HTT), also known as serotonin transporter (SERT), which actively transfers 5-HT back to 5-hydroxytryptamergic neurons. Dysregulation of the 5-HT system in patients with ASD is also accompanied by dysregulation of 5-HTT, but the exact relationship between 5-HTT changes and ASD remains to be determined. Currently, PET probes used in the study of ASD-related 5-HTT anomalies mainly include [^11^C](+)McN5652, [^11^C]DASB, [^18^F]Fmener-D2, and [^11^C]MADAM.

[^11^C](+)McN5652 was the first probe used to successfully image 5-HTT in the human body despite the relatively low signal-to-noise ratio and has a leading role in the field of 5-HTT imaging *in vivo* (Suehiro et al., 1993). Nakamura et al. (2010) explored the differences in 5-HTT between 20 adult males with high-functioning autism and 20 typical developing controls by using [^11^C](+)McN5652 PET. The results showed 5-HTT levels in the whole brain of men with autism were lower than in normal men. Compared to [^11^C](+)McN5652, [^11^C]DASB is more widely used and accepted due to its high repeatability and reliability that are a result of its high-affinity reversible binding with 5-HTT (Wilson et al., 2002). However, studies using [^11^C]DASB to detect 5-HTT in ASD have yielded different results. Girgis et al. (2011) conducted a [^11^C]DASB PET study on 17 adult patients with ASD and 17 typical developing controls, and found no statistically significant differences in 5-HTT between groups. More recently, Andersson et al. (2020) used [^11^C]MADAM PET to study 15 adult patients with autism and 15 typical developing controls, and found 5-HTT availability was significantly lower in the gray matter, brain stem, and nine other gray matter subregions in the group with autism. The results further support previous research suggesting that 5-HTT density is reduced in autism (Lundberg et al., 2006). Differences in conclusions of various studies may be a result of the heterogeneity of study subjects, which also suggests there may be differences in the molecular imaging manifestations in patients with different ASD subtypes.

In addition, some probes targeting 5-HTT (such as [^11^C]ADAM, [^11^C]DAPA, and [^11^C]AFM) have demonstrated their effectiveness in animal and human studies, which would promote the exploration of the relationship between 5-HTT and ASD, and improve our understanding of the pathophysiological mechanisms of ASD (Huang et al., 2001, 2002).

#### 5-Hydroxytryptamine Receptor

5-Hydroxytryptamine produces different physiological effects by stimulating different 5-HT receptor (5-HTR) subtypes. At present, 14 5-HTR subtypes have been found. The clear correlation between density changes in 5-HTR and ASD has not been clarified. By now, only the 5-HTR subtypes 5-HT_2A_R and 5-HT_1A_R have been used in ASD PET studies. The probes targeting 5-HT_2A_R include [^11^C]MDL100907, [^18^F]Setoperone, and [^18^F]Altanserin, and the probes targeting 5-HT_1A_R mainly include [^18^F]MPPF and [^18^F]F13714.

[^18^F]Setoperone is a reliable probe with high specificity for 5-HT_2A_R (Blin et al., 1990). Beversdorf et al. (2012) used [^18^F]Setoperone PET to study six patients with autism and 10 typical developing controls, and found there was less [^18^F]Setoperone binding in the thalamus of patients than in the control group with no significant differences in other regions. Goldberg et al. (2009) used [^18^F]Setoperone PET to study 19 parents of children with autism and 17 typical developing controls. The results showed that the cortical 5-HT_2A_R binding potential (BP_ND_) of the parents of the child with autism was significantly lower than that of the control group. Platelet 5-HT levels were significantly negatively correlated with the cortical 5-HT_2A_R BP_ND_ in the parents of the child with autism. The parents of the child with autism had low cortical 5-HT_2A_R density, consistent with reports of reduced 5-HT_2A_R expression and function in patients with ASD. It also further verified the pathophysiological mechanism of elevated 5-HT level in peripheral blood of ASD patients. In another study, the probe [^11^C]MDL100907 was used to characterize 5-HT_2A_R in AS. Imaging of 17 adult patients with AS and 17 typical developing controls showed that there was no statistical difference in regional [^11^C]MDL100907 BP_ND_ (Girgis et al., 2011).

To explore the relationship between 5-HT_1A_R binding potential, gray matter volume (GMV), and social personality, a PET study was performed on 18 adult patients with ASD (7 with Autism and 11 with AS) and 24 typical developing controls using a 5-HT_1A_R-specific probe [^18^F]MPPF (Lefevre et al., 2020). 5-HT_1A_R BP_ND_ had a regional negative correlation with GMV, although no difference in 5-HT_1A_R density was observed between the two groups. However, there were specific associations between 5-HT_1A_R, GMV, and social personality scores in the striatum of the control group not found in the ASD group. This suggests that there is a 5-HT system disturbance associated with 5-HT_1A_R density changes in the striatum of patients with ASD.

In addition to 5-HT_2A_R and 5-HT_1A_R, the application of PET probes targeting other subtypes of 5-HT receptors in ASD research can also be explored. Further probe development and quantitative imaging studies of 5-HTR will help improve our understanding of the pathogenesis of ASD (Zürcher et al., 2015).

### Oxytocin

Oxytocin (OXT) is a neuropeptide that plays an important role in the regulation of social behavior in mammals by interacting with oxytocin receptor (OTR). Previous studies have found abnormal OXT levels in patients with autism (Modahl et al., 1998). In addition, a large number of studies has confirmed intranasal OXT treatment can improve social behavior in autism. A study by Guastella et al. (2010) showed OXT treatment through intranasal inhalation could improve performance in an eye-mind task in those with autism. FMRI studies on children and adult patients with autism also found several brain regions, including the striatum, showed improved responses to stimuli after OXT treatment (Domes et al., 2013; Gordon et al., 2013).

Unfortunately, PET probes targeting OTR satisfactorily have yet to be successfully developed. The probes [^11^C]EMPA (Wang et al., 2013) and [^11^C]PF-3274167 (Vidal et al., 2017) have not been under continued use due to unsatisfactory physical and chemical properties. As OXT and 5-HT are structurally and functionally integrated, they collectively mediate emotion-based behavior. The amygdala is the center of OXT regulation of 5-HT. OXT can inhibit amygdala activity and decrease anxiety, while high amygdala activity and dysregulation of 5-HT are associated with increased anxiety. Therefore, some investigations about the mechanisms by which OXT plays a role in ASD by measuring changes in the human 5-HT system before and after OXT treatment were conducted. Mottolese et al. (2014) used [^18^F]MPPF PET to detect changes in 5-HT_1A_R in the brains of 24 healthy subjects taking OXT or a placebo, and found OXT increased [^18^F]MPPF BP_ND_ in the dorsal raphe nucleus (the core region of 5-HT synthesis), amygdala/hippocampus complex, insula, and orbitofrontal cortex. In a [^18^F]MPPF PET study of 18 adult patients with ASD and 24 typical developing controls, Lefevre et al. (2018) found no difference in the concentration and distribution of 5-HT_1A_R between patients with ASD and controls. However, OXT significantly increased [^18^F]MPPF BP_ND_ in some brain regions in the control group, but not in the ASD group. Hirosawa et al. (2017) imaged 10 adult males with autism men before and after OXT treatment using [^11^C]DASB PET. A significant increase in [^11^C]DASB BP_ND_ in the left inferior frontal gyrus was observed after OXT treatment and extended to the left middle frontal gyrus in those with autism. However, there was no significant correlation between changes in clinical symptoms and [^11^C]DASB BP_ND_. These studies revealed a partial role for OXT in inhibition of 5-HT signaling, as well as a partial relationship between changes in the serotonergic system and prosociality after OXT therapy. Further studies are needed to verify the underlying relationship between OXT and ASD.

### Dopamine System

As the most abundant catecholamine neurotransmitter in the brain, dopamine (DA) is involved in the regulation of various physiological functions of the central nervous system, including social reward and social motivation. A number of genetic studies on patients with autism have reported an association between DA transporter and receptor-associated gene mutations and ASD-associated clinical symptoms (Gadow et al., 2008; Staal et al., 2012; Hamilton et al., 2013). In addition, the efficacy of psychotherapeutic agents targeting the DA system in improving clinical symptoms in patients with ASD suggests that the DA system plays a role in mediating behavior in these patients. The DA system is an important focus of ASD neuroimaging research. There have been many PET studies on the DA system, but there is no consensus on the exact changes that occur for DA precursor, transporters, and receptors in the brains of patients with ASD.

#### Dopamine Precursor

Dopa is the precursor of DA, and can be absorbed, metabolized, and stored by dopaminergic endings. Imaging with [^18^F]FDOPA reflects DA synthesis levels in the brain. Ernst et al. (1997) studied the differences in brain DA synthesis between 14 children with autism and 10 neurotypical children using [^18^F]FDOPA PET. The DA ratios in the caudate nucleus, putamen, midbrain, and lateral and medial prefrontal regions (areas rich in dopaminergic endings) compared to the occipital cortex (area lacking dopaminergic endings) were calculated. The DA ratio in anterior medial prefrontal cortex/occipital cortex displayed a 39% decrease in those with autism compared with the neurotypical children. However, there were no significant differences in other measured areas. When [^18^F]FDOPA PET was used in eight adult patients with AS and five typical developing controls, the [^18^F]FDOPA inflow value (*K*i) in the striatum and frontal cortex in those with AS was increased compared the normal control group (Nieminen-von Wendt et al., 2004). However, a recent study comparing 44 adult patients with ASD and 22 typical developing controls using [^18^F]FDOPA PET did not find significant inter-group differences in the striatum’s ability to produce dopamine (Schalbroeck et al., 2021a,b). The results of these studies are inconsistent, which is most likely related to the heterogeneity of the study subjects.

#### Dopamine Transporter

In a previously mentioned study of 20 high-functioning adult patients with autism and 20 typical developing controls, DAT was measured using [^11^C]WIN-35,428 in addition to imaging 5-HTT using [^11^C]McN-5652. The results showed that patients with autism had significantly higher DAT binding in the orbital prefrontal cortex. In addition, DAT binding in the orbitofrontal cortex was inversely correlated with 5-HTT binding in those with autism (Nakamura et al., 2010).

#### Dopamine Receptor

Fernell et al. (1997) conducted a PET study on six children with autism before and after treatment with R-BH4, a cofactor of tyrosine hydroxylase in the catecholamine and serotonin biosynthesis pathways, using [^11^C]NMS. With improvement in symptoms, dopamine receptor 2 (D_2_R) in the caudate and putamen of children with autism was decreased by approximately 10% after R-BH4 treatment from abnormally high pre-treatment levels to normal levels. Fujino et al. (2020) investigated differences in the dopamine receptor 1 (D_1_R) between adults with autism and typical developing controls using [^11^C]SCH23390 PET, and found D_1_R binding in the striatum, anterior cingulate cortex, and temporal cortex was significantly negatively correlated with ASD detail attention scores and significantly positively correlated with emotional perception scores in those with autism. However, there was no significant intergroup differences in D_1_R binding in the striatum, anterior cingulate cortex, and temporal cortex. The latest study compared 10 adult patients with ASD with 12 typical developing controls using [^11^C]raclopride PET (Zürcher et al., 2021). Compared with the control group, the ASD group showed reduced D_2_R/D_3_R binding in bilateral putamen and left caudate nucleus.

### Amino Acid Neurotransmitters

Protein synthesis is necessary for many important processes in the brain, including synaptic plasticity, long-term memory, and experience-dependent development. Based in part on the association between ASD and single-gene mutations, such as in glial 3/4, neuroprotein 1, and shank 3, atypical synapse protein synthesis is thought to play a role in neurodevelopmental disorders, including ASD (Kelleher and Bear, 2008). Since amino acids are the basic components of proteins, the quantitative study of amino acids and its receptors has revealed some features of abnormal protein synthesis in ASD.

#### Gamma-Aminobutyric Acid

Gamma-aminobutyric acid (GABA) is a major inhibitory neurotransmitter in the brain and plays a role in synaptic pruning and regulation of development. GABA dysfunction may lead to an imbalance in excitation/inhibition of the nervous system, a phenomenon associated with ASD (Pizzarelli and Cherubini, 2011). Previous human genetics studies found mutations in the GABA_A_ receptor gene and genes related to GABA synthesis in those with autism (Cook et al., 1998; Coghlan et al., 2012). Autoradiography studies revealed reduced GABA_A_ in the hippocampus and anterior cingulate cortex of patients with autism (Blatt et al., 2001; Oblak et al., 2009). Studies on rodent models of ASD have found prolonged neuronal excitation caused by early GABA inhibition may be an underlying mechanism in ASD (Tyzio et al., 2014). Although a large body of evidence suggests changes in GABA may be associated with ASD, there are currently very few PET studies on GABA.

Mendez et al. (2013) used [^11^C]RO15-4513 PET to measure GABA_A_ receptor α1 and α5 subtype levels in three high-functioning adult patients with autism and three typical developing controls, and found that binding of [^11^C]RO15-4513 was significantly reduced in the brains of those with autism. Region of interest analysis also revealed significant reductions in the bilateral amygdala and NAc, a result associated with low levels of the GABA_A_ α5 subtype. These results provide preliminary evidence for the deletion of GABA_A_ α5 in autism and support the further study of ASD-related GABA system abnormalities. However, when [^18^F]FMZ was used in 15 adult patients with ASD and 15 typical developing controls, and [^11^C]RO15-4513 in 12 adult patients with ASD and 16 typical developing controls for PET, there were no differences found in the availability of GABA_A_ receptors or GABA_A_ α5 subtype in any region of the brain in adults with or without ASD (Horder et al., 2018). Fung et al. (2020) measured total GABA_A_ receptor densities in 28 adults patients with ASD and 29 typical developing controls using [^18^F]FMZ PET and GABA concentrations using ^1^H-MRS. Based on the bilateral thalamus and left dorsolateral prefrontal cortex (DLPFC) as the study areas, [^18^F]FMZ PET revealed no differences in GABA_A_ receptor density between the ASD and control groups. However, ^1^H-MRS measurements showed the GABA/Water ratio (GABA normalized to Water signal) in the left DLPFC of patients with ASD was significantly higher than in the control group. When it comes to the role of GABA in ASD pathogenesis, PET research still has a broad area to explore.

#### Glutamate

Based on the theory that there is an imbalance in brain excitation/inhibition in individuals with ASD, changes in glutamate are also an important focus of ASD neuroimaging research (Rubenstein and Merzenich, 2003). Currently, there are some probes with satisfactory radiochemical properties that can be applied to ASD studies on glutamate receptors, such as [^18^F]FPEB, [^11^C]ITMM, and [^11^C]MMPIP, which binds specifically to metabolic glutamate receptor 5 (mGluR_5_), metabolic glutamate receptor 1 (mGluR_1_), and metabolic glutamate receptor 7 (mGluR_7_), respectively (Barret et al., 2010; Sullivan et al., 2013; Toyohara et al., 2013; Wong et al., 2013). However, only [^18^F]FPEB has been used in clinical ASD PET research.

Metabolic glutamate receptor 5 visualization and animal model studies based on [^18^F]FPEB PET have confirmed the association between ASD and glutamate system imbalance. [^18^F]FPEB PET imaging of a Shank3 complete knockout mouse model (Shank3B-/-) showed that BP_ND_ of mGluR5 was significantly increased in the hippocampus, thalamus, striatum, and amygdala in Shank3B-/- mice compared with normal mice (Cai et al., 2019). Fatemi et al. (2018) used [^18^F]FPEB PET to study six adult patients with autism and three typical developing controls and found there were significantly higher [^18^F]FPEB BP_ND_ in the posterior central gyri and cerebellum of those with autism. There was a significant negative correlation between age and [^18^F]FPEB BP_ND_ in the cerebellum, but no significant negative correlation with the posterior central gyrus. Precuneus [^18^F]FPEB BP_ND_ was positively correlated with the sleepiness scale score on the Abnormal Behavior Checklist (ABC), while cerebellar [^18^F]FPEB BP_ND_ was negatively correlated with ABC total score, ABC hyperactivity subscale, and ABC inappropriate speech subscale. These new findings provide the first evidence that mGluR5 binding is altered in key regions of brains of those with autism, suggesting abnormal glutamate signaling in these regions. The correlations between [^18^F]FPEB binding potential changes in the cerebellum and precuneus suggest some ASD symptoms may be influenced by abnormal glutamate signaling. In a recent study, Brašić et al. (2021) used [^18^F]FPEB PET to compare the density and distribution of mGluR5 in the brains of patients with idiopathic autism spectrum disorder (IASD), fragile X syndrome (FXS), and TD. The results showed that the expression of mGluR5 in cortical regions of IASD patients was significantly increased compared with TD patients, while the expression of mGluR5 in all regions of FXS patients was significantly decreased compared with TD patients.

In addition, probes targeting mGluR1 and mGluR7 with good kinetic properties are currently under development and future imaging studies on ASD will reveal more about the pathophysiological mechanisms of glutamate’s role in ASD.

#### Leucine

Leucine content affects the rate of protein synthesis in neurons and has a physiologically significant role in regulation of dendritic spines, where defects in the latter are associated with neuropsychiatric diseases (Shih and Hsueh, 2018). Shandal et al. (2011) used [^11^C]Leucine PET to study overall brain protein synthesis in stunted children with and without PDD and measured the involvement of leucine in protein synthesis. It was determined that protein synthesis in the temporal lobe language region of stunted children with PDD was increased. Notably, previous cerebral perfusion imaging studies on patients with ASD also suggested there are abnormalities in the temporal lobe region and abnormal protein synthesis in the language region of children with developmental delay and PDD may be related to widespread symptoms. However, a new study used [^11^C] Leucine PET to measure cerebral protein synthesis in nine young patients with fragile X syndrome and 14 typical developing controls, no significant differences were found between the two groups (Schmidt et al., 2020). In addition, a robust radiolabeled assay was applied to measure rate of protein synthesis in peripheral blood mononuclear cells (PMCS) and platelets in 13 patients with fragile X syndrome and 14 typical developing controls by Dionne et al. (2021). The results showed that the protein synthesis rate was decreased in patients with fragile X syndrome. These findings challenge the reports of increased protein synthesis in fragile X syndrome. It indicates that more research is needed to explore the leucine abnormality associated with ASD.

#### Acetylcholine

Acetylcholinesterase (AChE) inhibitors have been reported to reduce aggression and inattention, as well as overall ASD symptoms in pharmacological studies and animal models of ASD (Hardan and Handen, 2002; Nicolson et al., 2006; Karvat and Kimchi, 2014). These studies provide evidence for the critical role of the cholinergic system in the etiology of ASD. However, there is currently only one PET study applied to these patients. When the acetylcholine analog [^11^C]MP4A was used to measure acetylcholinesterase activity, the main finding was a lack of cholinergic innervation in the fusiform gyri in adult patients with autism (Suzuki et al., 2011). Additional targeted PET studies in the future will help clarify the role of cholinergic dysfunction in the pathogenesis of ASD.

### Neuroinflammation

Immune-mediated mechanisms are considered to be among the pathophysiological factors leading to ASD (Stigler et al., 2009). Imaging studies, specimen studies, and animal models have revealed central nervous system inflammation, including microglial activation and underlying microglial pathology, in patients with neuropsychiatric disorders such as ASD, depression, and schizophrenia. In addition, various psychotropic drugs are thought to have a direct effect on microglia. However, the relationship between microglia, neurotransmitters, and neuropsychiatric disorders has not been fully elucidated to date (Kato et al., 2013).

Microglia play an important role in the growth and development of the central nervous system and immunity. Activated microglia overexpress the translocator protein (TSPO), which is the most common imaging biomarker for neuroinflammation. TSPO probes have been continuously in development, with the first generation being [^11^C]PK11195, the second generation [^11^C]DPA713, [^18^F]FEPPA, and [^11^C]PBR28, and the third generation [^11^C]ER176 and [^18^F]GE180 (Best et al., 2019; Werry et al., 2019). However, the use of these probes in ASD studies has been rarely reported. Using the first-generation TSPO probe [^11^C]PK11195, Suzuki et al. (2013) studied differences in microglia activation between 20 18- to 31-year-old high-functioning patients with ASD and 20 age and IQ matched controls. Increased binding of [^11^C]PK11195 in the fusiform gyrus, prefrontal cortex, cingulate cortex, midbrain and cerebellum was also reported in ASD patients. When 15 adult patients with ASD were compared with 18 typical developing controls using the second-generation TSPO probe [^11^C]PBR28, those with ASD were found to have lower regional TSPO expression in multiple brain regions, including the bilateral insular cortex, bilateral precuneus/posterior cingulate gyrus, bilateral temporal gyrus, angular gyrus, and superior limbic gyrus (Zürcher et al., 2020). The results of these two studies are inconsistent, which might be due to the heterogeneity of ASD patients, such as age, gender, IQ and subtype, and the influence of TSPO gene polymorphism in patients should also be considered. The efficacy of other second- and third-generation TSPO probes has been tested in studies of other neurological and psychiatric disorders and future imaging studies of patients with ASD will help to enrich our understanding of the pathophysiological mechanisms of ASD and understand disparities between previous studies (Owen et al., 2010; Fujita et al., 2017; Hafizi et al., 2017; Kobayashi et al., 2018).

**FIGURE 1:**
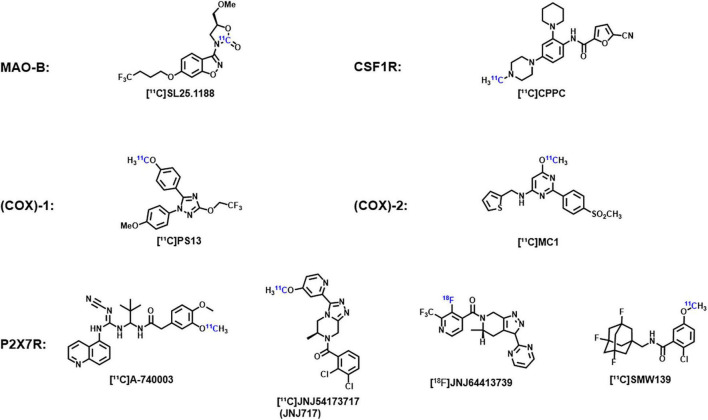
Chemical structures of the corresponding probes of monoamine oxidase B, cyclooxygenase-1, cyclooxygenase-2, colony-stimulating factor 1 receptors, and purinable P2X7 receptors.

In addition, due to the importance of neuroinflammation imaging, some promising new targets and corresponding probes are being explored. For example, monoamine oxidase B (MAO-B) and its specific probe [^11^C]SL25.1188, cyclooxygenase (COX)-1 and its specific probe [^11^C]PS13, cyclooxygenase (COX)-2 and its specific probe [^11^C]MC1, colony-stimulating factor 1 receptor (CSF1R) and its specific probe [^11^C]CPPC, and purinergic P2X7 receptor (P2X7R) and its specific probes [^11^C]JNJ-54173717 (JNJ-717), [^18^F]JNJ-64413739, and [^11^C]SMW139 (Figure 1). The key roles of these targets in neuroinflammation and the effectiveness of imaging using these probes have been demonstrated in previous experiments and their application will open up new directions in the study of neuroimaging of ASD (Meyer et al., 2020).

### Arginine Vasopressin

**FIGURE 2:**
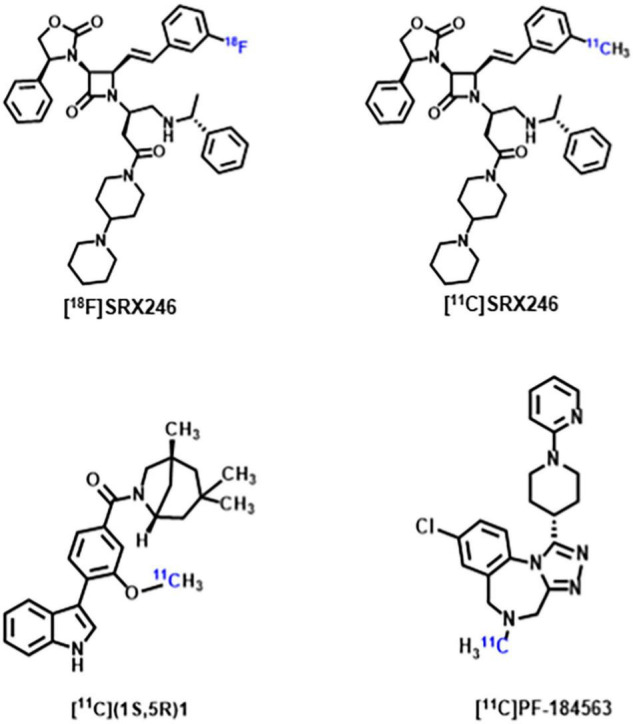
Chemical structure of V1aR targeting probes.

Arginine vasopressin (AVP) is a non-peptide neuroendocrine hormone synthesized by neurons in the hypothalamus. AVP interacts with the three G-protein-coupled receptors V1a/V1b/V2, and plays a variety of physiological roles in mammals including being an antidiuretic, increasing blood pressure, releasing adrenal corticosteroid, and regulating social behavior (Koob and Bloom, 1982; Jard, 1998). Since early animal studies have found AVP plays an important role in regulating biosocial behaviors, including mating, parentage, sociability and aggression, an increasing number of studies have focused on the correlation between AVP and ASD (Goodson and Thompson, 2010; Kelly and Goodson, 2014; Caldwell, 2017). Although no consistent conclusions have been reached regarding the differences in AVP concentrations between patients with ASD and normal controls, multiple human studies on the correlation between AVP and ASD suggest that AVP and its signaling pathways have a promising future in supporting ASD diagnosis and drug development (Miller et al., 2013; Shou et al., 2017; Parker et al., 2018; Wilczyński et al., 2019). Some recent studies have shown V1a receptor antagonists can modulate the action of AVP, thereby affecting the core symptoms of ASD. Balovaptan, a novel V1a receptor antagonist developed by Roche, showed improvement on the Vineland-II Adaptive Behavior Scale (including secondary endpoints of communication, social interaction and daily living skills) in a 12-week Phase 2 study of adults with ASD (Bolognani et al., 2019). The drug was therefore approved by the U.S. Food and Drug Administration in January 2018 for phase III clinical trials in adults with ASD and completed in July 2020. However, as of June 2020, balovaptan was discontinued in phase II clinical trials of ASD treatment in children and adolescents due to its ineffectiveness in this population (Schnider et al., 2020). Although the V1a receptor has been a new focus of ASD research, only [^18^F]SRX246 and [^11^C]SRX246 (Fabio et al., 2012), [^11^C](1S,5R)-1 (Naik et al., 2017), [^11^C]PF-184563 (Haider et al., 2021) PET probes targeting the V1a receptor have been recently developed (Figure 2). These PET probes have demonstrated their radiochemical properties in autoradiography and animal experiments, but have not yet been utilized in human experiments. The subsequent development of PET probes that can be applied to ASD studies will further reveal the role of AVP in the pathophysiological mechanisms of ASD.

## Conclusion

Autism spectrum disorder is a disease caused by multiple factors, both heredity and environmental, and its core cause remains unclear. Early diagnosis is very important for the treatment of ASD. Earlier and clearer diagnosis and intervention are conducive to improving the clinical symptoms of ASD in patients, which is an important influencer on treatment efficacy. Application of PET can dynamically and quantitatively evaluate resting and task states, cerebral glucose metabolism before and after treatment, cerebral blood flow perfusion, and neurotransmitter system biomarkers. It can also aid exploration of the pathophysiology of ASD and auxiliary of ASD diagnosis and treatment, and promote drug research and development. The discovery of ASD biomarkers and development of corresponding PET probes is the key to ASD PET research, which plays an important role in clinical diagnosis, drug development, and efficacy evaluation. Currently, studies have found biomarkers, such as those involved in glucose metabolism, cerebral blood perfusion, neurotransmitter systems (Table 4), and neuroinflammation, associated with ASD. However, ASD is a general designation of a group of neurodevelopmental disorders. Differences in subjects (e.g., age, gender, and IQ), experimental design (e.g., awake/sleep/anesthesia, resting-state/task-state, and imaging conditions), and different ASD subtypes may lead to contradictory conclusions among different studies (Lombardo et al., 2019). Although the clinical diagnostic criteria for autism, Asperger’s, atypical autism/unclassified pervasive developmental disorder, and disintegrated disorder in children are grouped into ASD for unified diagnosis and treatment based on behavioral definitions, the results of existing PET imaging studies suggest there may be differences in the pathophysiological mechanisms at the molecular level. For example, in PET studies of 5-HT and DA systems, imaging results of patients with AS are often inconsistent with those of patients with autism. This is a limitation of current cross-sectional studies on ASD, which may cause unexpected problems in subsequent research. Therefore, future ASD PET studies may be required to explore whether there are differences in the pathophysiological mechanisms in different subtypes of ASD at the molecular level. The results will serve as valuable references for clinical diagnosis and treatment of ASD, and will further guide the design and selection of experimental subjects, and maintain consistency in experimental methods in subsequent studies (Hong et al., 2020).

**TABLE 4:** Neurotransmitter and neuroinflammation PET studies in ASD.

System	Target	Citation	Tracer	Subject (*n*) Sex(M/F)	Age (year): mean ± SD, range	WAIS scores IQ: mean ± SD, range	Diagnosis	Conclusions
5-HT	Precursor	Chugani et al., 1997	[^11^C]AMT	ASD: *n* = 7(M)/1(F) CON: *n* = 4(M)/1(F)	ASD: 7, 4–11 CON: 10, 8–14	–	Autism [DSM-IV, GARS, CARS]	Abnormalities and asymmetries in dentate-thalamo-cortical pathway.
	Precursor	Chugani et al., 1999	[^11^C]AMT	ASD: *n* = 24(M)/6(F) Siblings: *n* = 6(M)/2(F) EP: *n* = 9(M)/7(F)	ASD: 6 ± 3, 2–15 Siblings: 9 ± 3, 2–14 EP: 6 ± 4, 1–13	–	Autism [DSM-IV, ADI-R, GARS, CARS]	Atypical age-related changes in 5-HT synthesis in ASD.
	Precursor	Chandana et al., 2005	[^11^C]AMT	ASD: *n* = 88(M)/29(F)	ASD: 7 ± 3, 2–15	–	Autism [DSM-IV, ADI-R, GARS, CARS]	Abnormal cortical asymmetry in ASD (64/117); Decreased 5-HT synthesis in ASD (58/64); Children with ASD and left hemispheric asymmetry more likely to have language impairments.
	5-HT_2_R	Goldberg et al., 2009	[^18^F]setoperone	Parents: *n* = 11(M)/8(F) CON: *n* = 8(M)/9(F)	Parents: 45 ± 6 CON: 44 ± 8	IQ: 64 ± 31	Autism [ADOS, ADI-R]	Decreased cortical 5-HT_2_R BPND in ASD; Platelet 5-HT levels negatively correlated with cortical 5- HT_2_ BPND.
	5-HTT DAT	Nakamura et al., 2010	[^11^C]McN-5652 [^11^C]WIN-35,428	ASD: *n* = 20(M) CON: *n* = 20(M)	ASD: 21 ± 2, 18–26 CON: 22 ± 2, 18–26	IQ: 99 ± 18, 71–140	Autism [DSM-IV-TR, ADI, ADOS]	Decreased 5-HTT in cingulate and thalamus and increased DAT in orbitofrontal cortex of ASD.
	5-HTT 5-HT_2A_R	Girgis et al., 2011	[^11^C]DASB [^11^C]MDL100907	ASD: *n* = 17(–) CON: *n* = 17(–)	ASD: 34 ± 11, 18–50 CON: 33 ± 10, 18–50	IQ: >85	AS [DSM-IV]	No differences in 5-HT_2A_ or 5-HTT BPND between groups.
	5-HT_2A_R	Beversdorf et al., 2012	[^18^F]setoperone	ASD: *n* = 5(M)/3(F) CON: *n* = 8(M)/4(F)	ASD: 31 ± 8 CON: 32 ± 10	FSIQ: 114 ± 15	Autism [DSM-IV, ADI-R]	Decreased 5-HT_2A_ in thalamus of ASD.
	5-HT_1A_R (OXT)	Mottolese et al., 2014	[^18^F]MPPF	24 health(M)	26 ± 6	–	–	5-HT_1A_R changes in the dorsal raphe nucleus correlated with OXT.
	5-HTT (OXT)	Hirosawa et al., 2017	[^11^C]DASB	ASD: *n* = 10(M)	ASD: 30 ± 6, 23–41	IQ: 102 ± 13, 85–130	Autism [DSM-IV-TR, ADOS]	Increased 5-HTT in left frontal gyrus correlated with OXT.
	5-HT_1A_R (OXT)	Lefevre et al., 2018	[^18^F]MPPF	ASD: *n* = 18(M) CON: *n* = 24(M)	ASD: 26 ± 6 CON: 34 ± 8	IQ: 100, 72–120	Autism or AS [DSM-IV-R, ADI]	Disturbed OXT and 5-HT_1A_R interaction in ASD.
	5-HTT	Andersson et al., 2020	[^11^C]MADAM	ASD: *n* = 11(M)/4(F) CON: *n* = 15(–)	ASD: 33 ± 9, 19–48 CON: 33 ± 9, 22–49	–	ASD [DSM-IV, ICD-10, ADOS]	Decreased 5-HTT in total gray matter, brainstem, and (9/18) examined subregions of gray matter.
	5-HT_1A_R	Lefevre et al., 2020	[^18^F]MPPF	ASD: *n* = 18(–) CON: *n* = 24(–)	ASD: 34 ± 8 CON: 26 ± 6	IQ: 100, 72–120	Autism or AS [DSM-IV, ADI]	Abnormal correlation between 5-HT_1A_R and GMV in left and right posterior putamen.
DA	Precursor	Ernst et al., 1997	[^18^F]FDOPA	ASD: *n* = 8(M)/6(F) CON: *n* = 7(M)/3(F)	ASD: 13 ± 2 CON: 14 ± 2	IQ: 74 ± 23, 46–123	Autism [DSM-III-R]	Decreased DA in mPFC of ASD.
	Precursor D_2_R	Fernell et al., 1997	[^18^F]FDOPA [^11^C]NMS	ASD: *n* = 4(M)/2(F)	ASD: 3–5	DQ: 20–70	Autism [DSM-III-R, CARS]	10% decreased D_2_R in caudate and putamen after R-BH4 treatment; No DA change after R-BH4 treatment.
	Precursor	Nieminen-von Wendt et al., 2004	[^18^F]FDOPA	ASD: *n* = 8(M) CON: *n* = 5(M)	ASD: 29 ± 6 CON: 31 ± 5	IQ: 113 ± 13, 97–140	AS [DSM-IV, ICD-10]	Increased DA in striatum and frontal cortex of ASD.
	Precursor	Schalbroeck et al., 2021a	[^18^F]FDOPA	ASD: *n* = 44(–) CON: *n* = 22(–)	ASD: 24 ± 3 CON: 23 ± 2	IQ: 104 ± 5	ASD [ADOS-2, AQ]	No differences between groups.
	D_2_R	Zürcher et al., 2021	[^11^C]raclopride	ASD: *n* = 10(M) CON: *n* = 10(M)/2(F)	ASD: 25 ± 4 CON: 26 ± 4	IQ:117 ± 12	ASD [DSM-V, ADOS-2]	Decreased phasic dopamine release to incentives in the bilateral putamen and left caudate
	D_1_R	Fujino et al., 2020	[^11^C]SCH23390	ASD: *n* = 18(M) CON: *n* = 20(M)	ASD: 33 ± 8 CON: 30 ± 6	IQ: 104 ± 16	Autism [DSM-IV-TR]	No differences between groups.
GABA	GABA_A_R α1, α5	Mendez et al., 2013	[^11^C]Ro15-4513	ASD: *n* = 3(M) CON: *n* = 3(M)	ASD: 34/41/43 CON: 37/39/40	FSIQ: 117–127	Autism [ICD-10, ADOS]	Decreased GABA_A_R in bilateral amygdala and nucleus accumbens of ASD.
	GABA_A_R α1, α5	Horder et al., 2018	[^11^C]FMZ [^11^C]Ro15-4513	(i) ASD: *n* = 11(M)/4(F) CON: *n* = 11(M)/4(F) (ii) ASD: *n* = 12(M) CON: *n* = 16(M)	(i) ASD: 33 ± 9 CON: 33 ± 9 (ii) ASD: 31 ± 9 CON: 30 ± 9	(i) FSIQ: 107 ± 18 (ii) FSIQ: 116 ± 14	ASD [DSM-V, ADOS, ADI-R, ICD-10]	No differences between groups.
	GABA_A_R	Fung et al., 2020	[^18^F]FMZ	ASD: *n* = 17(M)/11(F) CON: *n* = 19(M)/10(F)	ASD: 27 ± 8 CON: 27 ± 7	FSIQ: 102 ± 17 VIQ: 104 ± 18 NVIQ: 100 ± 1.5	ASD [DSM-V, ADI-R, ADOS-2]	No differences between ASD and TD in bilateral thalami and DLPFC.
Glutamate	mGluR5	Fatemi et al., 2018	[^18^F]FPEB	ASD: *n* = 6(M) CON: *n* = 3(M)	ASD: 20 ± 2 CON: 27 ± 4	–	Autism [DSM-IV, SCID-CV, ADOS, ADI-R, ASSQ, CGI, ABC, SCQ, GAF]	Altered mGluR5 in precuneus, postcentral gyrus and cerebellum of ASD.
	mGluR5	Brašić et al., 2021	[^18^F]FPEB	IASD: *n* = 6(M)/1(F) FXS: *n* = 10(M) TD: *n* = 10(M)/9(F)	ASD: 20 ± 2 FXS: 30 ± 10 TD: 35 ± 15		IASD [DSM-V, ADI-R, ADOS]	↑ mGluR5 in cortical regions of IASD and ↓mGluR5 in all regions of FXS.
ACh	AChE	Suzuki et al., 2011	[^11^C]MP4A	ASD: *n* = 14(M)/6(F) CON: *n* = 14(M)/6(F)	ASD: 24 ± 4, 18–33 CON: 23 ± 4, 19–32	FSIQ: 92 ± 20, 70–140	Autism [DSM-IV-TR, ADI-R, ADOS]	Decreased AChE in bilateral fusiform gyri, and negatively correlated w/social disabilities.
Leucine	Leucine	Shandal et al., 2011	[^11^C]leucine	DD + PDD: *n* = 7(M)/1(F) DD - PDD: *n* = 5(M)/3(F)	DD + PDD: 6 ± 2 DD - PDD: 7 ± 1	–	DD ± PDD [GARS, VABS]	Increased protein synthesis rate in left posterior middle temporal area in DD w/PDD, also correlated w/GARS scores.
	Leucine	Schmidt et al., 2020	[^11^C]leucine	FXS: *n* = 9(M) CON: *n* = 14(M)	FXS: 21 ± 1, 18∼24 CON: 22 ± 1, 18∼24	–	DNA analysis SCID-I/NP	No protein synthesis change in brain
Neuro-inflammation	TSPO	Suzuki et al., 2013	[^11^C]PK11195	ASD: *n* = 20(M) CON: *n* = 20(M)	ASD: 23 ± 5, 18–35 CON: 23 ± 4, 19–32	FSIQ: 96 ± 17, 81–140	ASD [DSM-IV-TR, ADI-R, ADOS]	Increased TSPO expression in fusiform gyrus, prefrontal cortex, cingulate cortex, midbrain and cerebellum
	TSPO	Zürcher et al., 2020	[^11^C]PBR28	ASD: *n* = 15(M) CON: *n* = 18(M)	ASD: 24 ± 6 CON: 26 ± 6	IQ: 86 ± 19	ASD [DSM-IV-TR, ADI-R, ADOS]	Lower TSPO expression in bilateral insular cortex, bilateral precuneus/posterior cingulate gyrus, bilateral temporal gyrus, angular gyrus, and superior limbic gyrus

*ACH, acetylcholine; AChE, acetylcholinesterase; 5-HT, serotonin; 5-HT_2A_R, serotonin 2A receptor; 5-HTT, serotonin transporter; ABC, Aberrant Behavior Checklist; AChE, acetylcholinesterase; ADI, Autism Diagnostic Interview; ADI-R, ADI-revised; ADOS, Autism Diagnostic Observation Schedule; ASD, autism spectrum disorder; ASSQ, Autism Spectrum Screening Questionnaire; AQ, Autism Spectrum Quotient; BA, Brodmann area; CARS, Childhood Autism Rating Scale; CGI, Clinical Global Impression; D_2_R, dopamine D2 receptor; DA, dopamine; DAT, dopamine transporter; DD, developmental delay; DQ, Developmental Quotient; DSM-III, Diagnostic and Statistics Manual of Mental Disorder Third Edition; DSM-III-R, DSM Third Edition Revised; DSM-IV, DSM Fourth Edition; DSM-IV-TR, DSM Fourth Edition Text Revised; DSM-5, DSM Fifth Edition; EP, epilepsy; FISQ, Full Scale Intelligence Quotient; FXS, fragile X syndrome; GABA_A_R, γ-aminobutyric acid type A receptor; GAF, Global Assessment of Functioning; GARS, Gilliam Autism Rating Scale; GMR, glucose metabolic rate; HFA, high-functioning autism; IASD, idiopathic autism spectrum disorder; ICD-10, International Classification of Diseases Tenth Revision; ICDS, Interview for Childhood Disorders and Schizophrenia; IQ, Intelligence Quotient; mPFC, medial prefrontal cortex; NVIQ, Non-verbal Intelligence Quotient; PDD, pervasive developmental disorder; PIQ, Performance Intelligence Quotient; R-BH4, 6R-L-erythro-5,6,7,8-tetrahydrobiopterin; SCID, Structured Clinical Interview; SCQ, Lifetime Social Communication Questionnaire; TSPO, translocator protein; VABS, Vineland Adaptive Behavior Scale; VIQ, Verbal Intelligence Quotient.*

In this paper, we summarize several targets, including cerebral glucose metabolism, cerebral blood perfusion, neurotransmitter system, neuroinflammation, arginine vasopressin receptor, as well as the corresponding positron probe and existing research results, which can be applied in ASD imaging studies. It can be seen that due to the repeated emphasis on the heterogeneity of ASD patients, PET studies using different probes can find some common results, while PET studies using the same probe will also produce different results. Just as TSPO gene polymorphism affects the imaging efficiency of different PET probes, the heterogeneity of ASD patients also strongly affects the consistency of probe imaging results for each target (Owen et al., 2012). Therefore, although it is important to explore new biomarkers and develop PET probes for ASD research, it is difficult to evaluate which probes have more application value in the existing system. Rather than using a probe to look for common results within the broad basket of ASD, it is more valuable to look for and establish a specific association between a biomarker, a probe, and a specific subtype of ASD at the molecular level would be more valuable (McPartland et al., 2020). This is the biggest challenge for ASD positron probe development and a more valuable exploration direction.

In-depth studies based on PET molecular imaging can provide a new perspective for the diagnosis and treatment of ASD, but there are also some difficulties. PET/CT and PET/MR imaging require patients to lie on a machine for scanning after injection of the probe. The whole imaging process requires protection from some radiation, long preparation and scanning period, and high coordination of patients. ASD usually presents during childhood, besides, patients have a large age span and significant differences in IQ. To carry out resting or task state PET imaging research, it is often necessary for professional technicians to carry out targeted training on patients, including on drug injection, adaptation to the scanning environment, and familiarity with the task process and, if necessary, anesthesia operation. These problems are not necessary to overcome for ASD experimental research, but also for clinical practice. It requires the joint efforts of doctors, technicians, nurses, and researchers in nuclear medicine, neuropsychiatry, rehabilitation, and other specialties to promote continuous advancements in ASD diagnosis and treatment research.

### Limitations

This paper mainly focused on the PET imaging research in neuroimaging of ASD, including the targets and corresponding PET probes. However, ASD is a general term for a group of developmental disorders. The time span of literature research is long, the difference in diagnostic criteria is large, and the heterogeneity of research objects is obvious. These factors lead to the poor comparability of the conclusions from the relevant imaging studies. In addition, the reliability of PET methodology and imaging efficiency of various PET probes (i.e., time window for data acquisition, *in vivo* specificity, test–retest reliability) are also important factors leading to the confusion about the current ASD research results. In the future, categorizing and analyzing the sample size, age, gender, IQ, ASD subtype and other influencing factors of subjects by single factor control will be conducive to better understanding the research outcomes and exploring new research ideas. In addition, the research results only relying on one neuroimaging technique are limited, and the combination of multiple imaging modalities is a more valuable direction.

## Author Contributions

ZT, HW, and XS contributed to the writing of the manuscript. WM and JY collect and sort out literatures. WY and XL contributed to the English language editing. LH and SZ constructed the figures and tables. SY provided constructive suggestions. HX and LW conceived the project and modified the manuscript. All authors contributed to the article and approved the submitted version.

## Conflict of Interest

The authors declare that the research was conducted in the absence of any commercial or financial relationships that could be construed as a potential conflict of interest.

## Publisher’s Note

All claims expressed in this article are solely those of the authors and do not necessarily represent those of their affiliated organizations, or those of the publisher, the editors and the reviewers. Any product that may be evaluated in this article, or claim that may be made by its manufacturer, is not guaranteed or endorsed by the publisher.
